# Non-Mutated Nucleophosmin 1 Is Recognized by the CD8+ T Lymphocytes of an AML Patient after the Transplantation of Hematopoietic Stem Cells from an HLA-Haploidentical Donor

**DOI:** 10.3390/curroncol29050239

**Published:** 2022-04-20

**Authors:** Sarka Nemeckova, Kamila Alexova-Zurkova, Petr Hainz, Jitka Krystofova, Jana Mackova, Katerina Roubalova, Marketa Stastna-Markova, Milena Vrana, Jan Vydra

**Affiliations:** 1 Institute of Haematology and Blood Transfusion, CZ-128 20 Prague, Czech Republic; kamila.zurkova@uhkt.cz (K.A.-Z.); petr.hainz@uhkt.cz (P.H.); jitka.krystofova@uhkt.cz (J.K.); jana.mackova@uhkt.cz (J.M.); katerina.roubalova@uhkt.cz (K.R.); marketa.markova@uhkt.cz (M.S.-M.); milena.vrana@uhkt.cz (M.V.); jan.vydra@uhkt.cz (J.V.); 2 Institute of Clinical and Experimental Hematology, 1st Faculty of Medicine, Charles University, CZ-121 08 Prague, Czech Republic

**Keywords:** acute myeloid leukemia, T cell response, nucleophosmin 1, mutation, hematopoietic stem cell transplantation

## Abstract

**Simple Summary:**

Our study describes an AML patient whose leukemia cells carried the *NPM1c^+^* mutation, and who was the recipient of allogeneic HSCT from a haploidentical donor. The patient raised a robust allorestricted CD8^+^ T cell response directed against the NPM1^wt^ protein. Favourably, the response against NPM1^wt^ was not accompanied by side effects such as GvHD. Moreover, the induction of a high NPM1^wt^ specific response coincided with the decrease in NPM1c^+^ transcripts detected, implying a beneficial graft versus leukemia effect. On the basis of these results, we suppose that TCRs from allorestricted NPM1^wt^-specific T cells are worth studying in other recipients of grafts from haploidentical donors as a possible tool for TCR gene therapy.

**Abstract:**

Nucleophosmin (NPM1, B23) is a multifunctional phosphoprotein expressed in all tissues. The protein is mainly localized in nucleoli. In hematological malignancies, *NPM1* belongs to commonly altered genes. Its mutation, always heterozygous, leads to the re-localization of the NPM1 protein from the nucleolus to the cytoplasm (NPM1c^+^). NPM1c^+^ is found in 30% of acute myeloid leukemia (AML). Our study showed that an AML patient, whose leukemia cells carried the *NPM1c^+^* mutation and who was the recipient of allogeneic HSCT from a haploidentical donor, raised a robust allorestricted CD8^+^ T cell response directed against the NPM1^wt^ protein. Favourably, the response against NPM1^wt^ was not accompanied by side effects such as GvHD. Moreover, the induction of a high NPM1^wt^-specific response coincided with the decrease in NPM1c^+^ transcripts detected, implying a beneficial graft versus leukemia effect. On the basis of these results, we suppose that TCRs from allorestricted NPM1^wt^-specific T cells are worth studying in other recipients of grafts from haploidentical donors as a possible tool for TCR gene therapy.

## 1. Introduction

Nucleophosmin (NPM1, B23) is a multifunctional phosphoprotein (37 kDa), expressed ubiquitously in all tissues. The protein is mainly localized in nucleoli and rapidly travels between the nucleus and the cytoplasm. NPM1 is involved in multiple cellular functions (for review see Falini B et al. [1]), such as ribosome assembly and exportcentrosome duplication and cell cycle control, DNA repair, histone- and protein-chaperone activity, 2-O methylation of rRNA, and response to stress stimuli. NPM1 is overexpressed in many types of solid human cancers. In some cases, meta-analyses have indicated that NPM1-increased expression correlates with the mitotic index and with the stage of tumor progression, and may act as a valuable prognosis biomarker and a potential therapeutic target in human solid tumors [2,3].

In hematological malignancies, *NPM1* belongs to commonly altered genes. Its mutation is always heterozygous and causes a dominant negative phenotype. In 30% of patients with acute myeloid leukemia (AML), *NPM1* mutations typically consist of 4-base pair frameshift duplication or insertion within exon 12 of the gene [4,5]. The frameshift results in the substitution of 7 C-terminal amino acids for a new 11 amino acid sequence. This leads to the loss of the nucleolar localization signal (NoLS) and the generation of a novel nuclear export signal (NES), which re-localizes the NPM1 mutant protein from the nucleolus to the cytoplasm (NPM1 cytoplasmic or NPM1c^+^). The oligomerization of NPM1c^+^ with non-mutated nucleophosmin (NPM1^wt^) results in the delocalization of the majority of the latter and in a reduction in NPM1^wt^ content in the nucleoli [1].

The sequence of 11 residues of the C-terminus of all types of mutated NPM1 proteins code for several unique oligopeptides that have been shown to function as leukemia-specific neoantigens. Several peptide epitopes derived from the C-terminal-mutated sequence were predicted in silico, and their function as ligands of specific T cells have been confirmed in vitro and in vivo. For a complete list of epitopes, see Table 1 in the review of Forghieri et al. [6]. It was shown that the frequency of specific T-cell responses against the mutated C-terminus differed significantly between AML patients with *NPM1c^+^* and healthy volunteers [7].

Previous studies have shown that the NPM1^wt^ protein is also immunogenic. Chronic graft versus host reaction in patients after bone marrow transplantation elicited antibodies specific for the NPM1^wt^ [8]. Cytotoxic T-lymphocyte (CTL) lines recognizing the NPM1^wt^ protein sequences which were able to lyse tumor cells overexpressing NPM1^wt^ were derived from patients with colorectal cancer [9]. It has been shown in several studies of HLA ligandome [10,11,12] that peptides from cleaved NPM1^wt^ are frequently found in complex with HLA in healthy blood cells and tumor cells, thus contributing to its role as a target for specific T cells. Whereas the NPM1c^+^ neoantigen may be considered an ideal targetable antigen for AML immunotherapy, the NPM1^wt^, under normal conditions, induces tolerance to the autoantigen by deletion of specific T cell clones. However, in the presence of detrimental allo-HLA immune responses after the transplantation of HLA-mismatched stem cells, T cells with high avidity for other autoantigens can be isolated from patients, as has been shown for the PRAME antigen [13]. Similarly, high-avidity T cells were induced by ex vivo stimulation with tumor antigens on HLA-mismatched antigen-presenting cells [14,15,16].

The aim of our study was to analyze the T cell response specific for both mutated NPM1c+ and NPM1^wt^ elicited in a hematopoietic stem cell transplant (HSCT) recipient with AML harboring *NPM1c^+^* mutation.

## 2. Material and Methods

### 2.1. Patient and Donor

A 64-year-old man (ID 1515) with acute myeloid leukemia with NPM1 mutation underwent HSCT from a haploidentical related donor, as a treatment of a molecular relapse of AML after his first complete remission. Genetic analysis at the time of diagnosis revealed normal karyotype, positivity for NPM1-type A, and DNMT3A mutations. At the time of transplantation, bone marrow examination showed morpholologic CR with minimal residual disease detected with flow cytometry (LAIP 0.04, LSC below the detection limit), and RT-PCR (NPM1c^+^ level was 2 copies/10^4^ abl mRNA).

**Figure 1:**
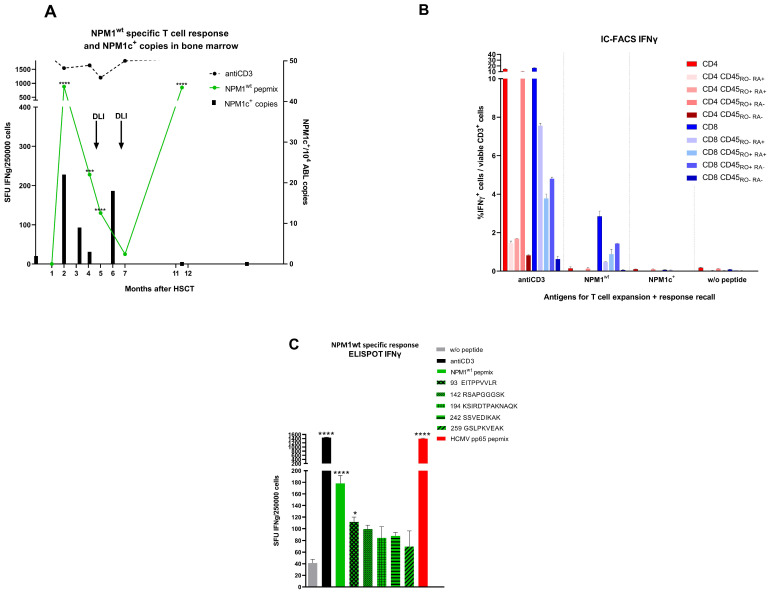
Nucleophosmin-specific T cell responses in an AML patient after HSCT. (**A**) Frequency of spot-forming units (SFU) specific for NPM1^wt^ detected by ELISPOT-IFNγ and result of quantification of NPM1c^+^ transcripts in bone marrow. Time of DLI is denoted by arrows. (**B**) The phenotype of NPM1^wt^-specific T cells at month +3 producing IFNγ determined by IC FACS. (**C**) Frequency of T cells specific for CTL epitopes 93-EITPPVVLR, 142-RSAPGGGSK, 194-KSIRDTPAKNAQK, 242-SSVEDIKAK, and 259-GSLPKVEAK determined at month +5. Statistical analysis was performed using the Mann–Whitney test (**A**,**C**) **** *p* < 0.0001, *** *p* < 0.001, * *p* < 0.05.

The patient’s HLA typing was: HLA-A*11:01; *23:01, HLA-B*13:02; *35:01, HLA-C *04:01; *06:02; DRB1 *01:01; *07:01, DQB1*05:01; *02:02; DPB1*04:01; *17:01. His 39-year-old son, who was HLA-genotypically haploidentical to the patient (HLA-A*23:01; *32:01, HLA-B*13:02; *15:01, HLA-C*03:03; *06:02, DRB1 *07:01; *08:03, DQB1 *02:02, *03:01, DPB1 *04:02; *17:01), was selected to serve as the HSC donor. The monitoring of NPM1c^+^ transcripts revealed a molecular relapse in bone marrow two months after HSCT (increase in the amount of NPM1c^+^ transcripts from 2 to 22 copies/10^4^ abl, Figure 1A). The signal of NPM1c^+^ was never found in the peripheral blood. The patient received pre-emptive donor lymphocyte infusions (DLI) (3 × 10^5^/kg and 1 × 10^6^/kg) on day 135 and 166, respectively, which resulted in a drop in NPM1c^+^ positivity. The absence of NPM1c^+^ transcripts in bone marrow lasted for a subsequent 17 months. The patient developed no severe complications, no GvHD or microbial infections. The patient was enrolled in an institutional clinical study to monitor the reconstitution of the immune system after transplantation. The patient signed an informed consent form. The study was approved by the institutional ethical board.

### 2.2. Detection of LAA-Specific T Cells

The isolation of PBMCs from patient blood and the measurement of LAA-specific T cell responses by ELISPOT-IFNγ and IC-FACS have been described in detail recently [17]. In brief, the patient’s PBMCs were pulsed with peptide pools and cultured in vitro in the presence of IL4 and IL7 for 11 days. Thereafter, the antigen-specific recall (PepMix 1 ug/mL) response of T cells was detected by ELISPOT-IFNγ or by IC-FACS. Pools of 15 amino acid long overlapping peptides (PepMix^TM^) covering the full-length sequence of WT1, PRAME, MUC1, CCNA1, NPM1^wt^ protein, and the C-terminal region of the NPM1c^+^ type A were purchased from JPT Peptide Technologies GmbH (Berlin, Germany). Five candidate NPM1^wt^ CTL epitopes, 93-EITPPVVLR, 142-RSAPGGGSK, 194-KSIRDTPAKNAQK, 242-SSVEDIKAK, and 259-GSLPKVEAK, restricted by HLA-A*11:01, were predicted using the Syfpeithi [18] and NetCTLpan [19] algorithms and synthesized by JPT Peptide Technologies GmbH.

## 3. Results

**Figure 2:**
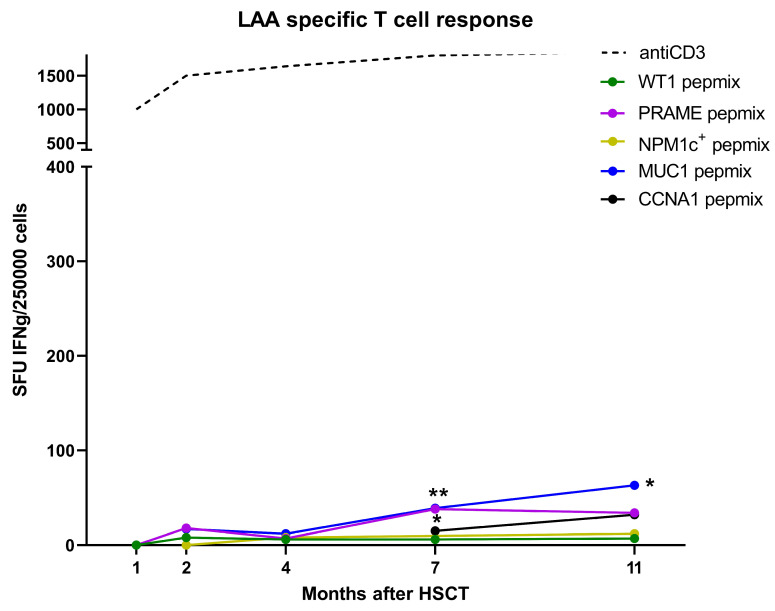
Monitoring of LAA-specific T cell responses in an AML patient after HSCT. Frequency of SFU specific for Wilm´s tumor antigen (WT-1), PRAME, mutated NPM1c^+^, mucin (MUC-1) and cyclin A1 (CCNA1) was detected by ELISPOT-IFNγ. Statistical analysis was performed using the Mann–Whitney test ** *p* < 0.01, * *p* < 0.05.

The patient (ID 1515) took part in an institutional clinical study monitoring the reconstitution of the T cell response against LAA, including WT1, PRAME, NPM1^wt^, NPM1c^+^, MUC1, and CCNA1. The response was measured at post-HSCT months 1, 2, 3, 4, 5, 7, and 11 by ELISPOT-IFNγ (Figure 1A, Figure 2). Antigen-specific T cell response against any LAA was entirely negative at post-HSCT month +1. Then, the T cell response against NPM1^wt^ (Figure 1A) rose enormously to 880 spots per 2.5 × 10^5^ cells at month +2 and remained significantly positive at months +4 and +5. Subsequently, it gradually declined and there was no significant difference in the response between NPM1^wt^-stimulated and unstimulated T cells at month +7. Later on, a high number of 800 spots appeared again at month +11. Concerningly, T cells against other LAA (Figure 2) with spots specific for PRAME and MUC1 were found at months +7 and +11. It should be noted that the decline in NPM1c^+^ transcript level had already started before administration of the first DLI; hence, it was associated with the presence of NPM1^wt^-specific T cells. The detection of intracellular IFNγ by IC-FACS (Figure 1B) revealed that the NPM1^wt^-responding T cell population at month +3 consisted mainly of CD8^+^ T cells, 80% of which were of the memory CD45RO^+^ phenotype. Our next goal was to identify epitopes recognized by the patient’s T cells. PBMCs collected at month +5 were pulsed with NPM1^wt^ PepMix, expanded for 11 days in vitro, and thereafter restimulated with NPM1^wt^ peptide MHC I epitopes. Measurement of the response by ELISPOT-IFNγ (Figure 1C) showed that the 93-EITPPVVLR epitope was recognized by T cells. The response elicited by other peptides was lower and insignificant in comparison with unstimulated cells.

## 4. Discussion

In this study, we monitored the response of T cells against LAA in a transplanted AML patient whose leukemia cells carried the *NPM1c^+^* (type A) mutation. A mighty response of CD8^+^ T cells directed against the NPM1^wt^ protein was found between months +2–+5 and at +11 month after transplantation, and coincided with an early molecular relapse detected as an increase in NPM1c^+^ transcripts in bone marrow between months +2 and +6. When compared with other LAA, the T cell response of the presented patient against NPM1^wt^ was much higher and differed significantly by frequency, phenotype, and dynamics. Unlike T cells against other LAA, which were mostly CD4^+^ (not shown), NPM1^wt^-specific T cells in this case were of the CD8^+^ phenotype. The peak of anti-NPM1^wt^ response (880 spot forming units (SFU)) was detectable as early as month +2 after transplantation, which may imply that the response was mediated by allorestricted memory T cells of donor origin. This presumption was confirmed by finding a positive IFNγ response after restimulation with minimal epitope 93- EITPPVVLR, which was predicted to be efficiently recognized in the context of the mismatched HLA-A*11:01 molecule of the recipient. The high frequency of NPM1^wt^-specific T cells may suggest that they were memory T cells generated by the donor in response to a heterologous antigen and that, after transplantation, they cross-reacted with NPM1^wt^ peptides derived from AML blasts in the context of the allo-HLA-A*11:01 molecule. The T cells which recognize NPM1^wt^- derived peptides in the context of their own HLA should be clonally deleted in healthy subjects. However, due to molecular mimicry, T cells of other specificity may recognize them under allogeneic conditions. The mechanism resembles the phenomenon called heterologous immunity [20], as observed in virus-infected kidney transplant recipients. It was shown [21,22] that, in those patients, HCMV-specific CD8(+) T cells can cross-react with alloantigens presented by mismatched or matched HLA molecules. Such cross-reactive T cells may damage the transplanted organ by a strong immune reaction. Notably, these cross-reactive T cells were detectable in the first few weeks following transplantation, but could not be detected in later months [21], thus resembling the kinetics of NPM1^wt^-specific T cells seen in our patient. A similar immune response was reported by Amir et al. [13] in association with hematopoietic stem cell transplantation. The authors isolated a clone of high-avidity allo-HLA-restricted T cells specific for tumor associated antigen PRAME from an AML patient with GvHD after the transplantation of stem cells from a mismatched donor. Moreover, some authors speculate that upregulation and subcellular delocalization of the complex in AML blasts containing the mutated NPM1c^+^ molecule could enhance immunogenicity of the common NPM1^wt^ sequence [23]. Another explanation of our results could be the NPM1c^+^-associated modulation of antigen processing machinery such as proteasome [24], immunoproteasome, or autophagy [25] involved in protein cleavage and peptide transport, and/or MHC I complex formation, and/or the modification of these antigen-presenting functions resulting from the different sensitivities of NPM1c^+^ cells to proinflammatory cytokines [26]. All these hypotheses have yet to be experimentally tested.

Surprisingly, we did not detect T cell response against NPM1c^+^-derived neoepitopes in our patient. Either these epitopes could not be presented in the context of the donor HLA, or the priming of such a response under highly immunosuppressive conditions during the first months after HSCT was ineffective. Minimal epitopes used in our study were predicted in silico as binders of HLA-A*11:01. Two of them, 93- EITPPVVLR and 194-KSIRDTPAKNAQk, were also detected as the true ligands of HLA-A*11 molecules on AML blasts [12], various other malignant cells, and the leukocytes of healthy subjects [10,11].

## 5. Conclusions

Our study has shown, for the first time, that an AML patient can mount a mighty T cell response against an autoantigen, the NPM1^wt^, under conditions of allogeneic HSCT from a haploidentical donor. Favorably, the allorestricted response against NPM1^wt^ persisting for 11 months was not accompanied by side effects such as GvHD during this period. Moreover, the induction of a high NPM1^wt^-specific response coincided with the decrease in NPM1c^+^ transcripts detected, implying a beneficial graft versus leukemia effect. On the basis of these results, we suppose that TCRs from allorestricted NPM1^wt^-specific T cells are worth studying in other recipients of graft from a haploidentical donor as a possible tool for TCR gene therapy.

## Data Availability

All data relating to this study are reported in the manuscript and figures.
